# Massive tricuspid regurgitation caused by iatrogenic papillary muscle rupture during leadless pacemaker retrieval: a case report

**DOI:** 10.1093/ehjcr/ytag418

**Published:** 2026-06-09

**Authors:** Hikaru Tanemura, Noriaki Moriyama, Masato Murakami, Shigeru Saito

**Affiliations:** Department of Cardiology, Shonan Kamakura General Hospital, Okamoto 1370-1, Kamakura, Kanagawa 247-8533, Japan; Department of Cardiology, Shonan Kamakura General Hospital, Okamoto 1370-1, Kamakura, Kanagawa 247-8533, Japan; Department of Cardiology, Shonan Kamakura General Hospital, Okamoto 1370-1, Kamakura, Kanagawa 247-8533, Japan; Department of Cardiology, Shonan Kamakura General Hospital, Okamoto 1370-1, Kamakura, Kanagawa 247-8533, Japan

**Keywords:** Leadless pacemaker, Papillary muscle rupture, Tricuspid regurgitation, Case report

## Abstract

**Background:**

Leadless pacemakers (LPMs) reduce many complications associated with transvenous systems; however, tricuspid regurgitation (TR) caused by mechanical interference after LPM implantation has been reported. We describe a rare case of massive TR caused by papillary muscle rupture during LPM deployment and retrieval.

**Case summary:**

An 86-year-old man presented with syncope due to complete atrioventricular block. Because of his advanced age and limited activities of daily living, a leadless pacemaker was selected. During deployment, the anterior papillary muscle became entrapped between the delivery sheath and the leadless pacemaker during deployment. Subsequent retrieval resulted in papillary muscle rupture and severe TR. The patient later underwent transvenous pacemaker implantation and recovered with medical therapy. He was transferred to a rehabilitation hospital without requiring additional intervention for tricuspid valve regurgitation.

**Discussion:**

This case highlights a previously unreported mechanism of TR during LPM implantation—papillary muscle entrapment and rupture during device retrieval—which can lead to catastrophic tricuspid valve dysfunction.

Learning point

- Acute papillary muscle rupture is a rare but catastrophic complication of leadless pacemaker retrieval.
- Entrapment of subvalvular structures during recapture should be suspected when resistance is encountered during device retrieval.
- Pre-procedural assessment of right ventricular anatomy may help identify patients at increased procedural risk.

## Introduction

Leadless pacemakers (LPMs) have demonstrated superiority over transvenous pacemakers (TV-PMs) in reducing complications such as infection, haematoma, and lead-related failure.^1,2^ However, mechanical interference with the tricuspid valve after LPM implantation has been associated with the development of tricuspid regurgitation (TR).^3,4,5^ In addition, reports of iatrogenic TR caused by tricuspid valve injury during LPM implantation remain limited. We present a case of massive TR caused by rupture of the anterior papillary muscle during LPM implantation.

## Summary figure

**Figure:**
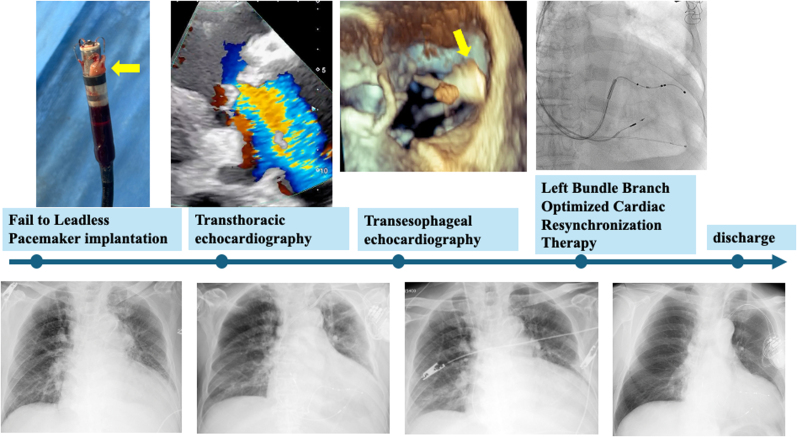


## Case presentation

An 86-year-old man presented to the emergency department following a syncopal episode accompanied by head trauma. Electrocardiography revealed complete atrioventricular block with a ventricular rate of 39 bpm. Chest radiography demonstrated cardiomegaly with mild pulmonary congestion (*Figure 1*). Transthoracic echocardiography (TTE) showed diffuse hypokinesis of the left ventricular. Although TV-PM implantation was initially considered, LPM (Micra AV; Medtronic, Minneapolis, MN, USA) was selected because of his advanced age and limited functional status.

**Figure 1:**
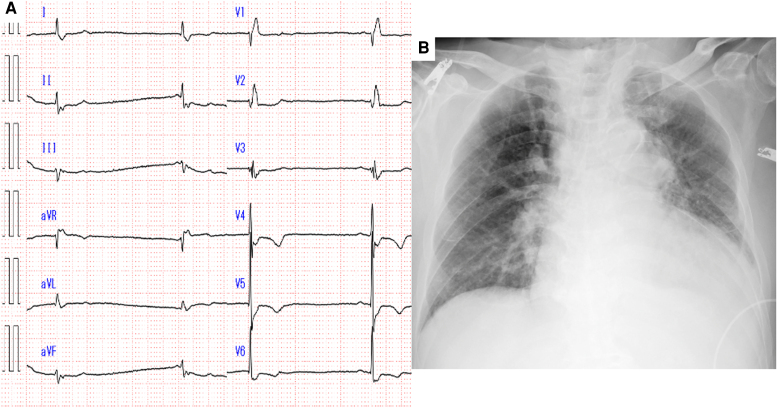
12-lead electrocardiogram and chest radiography. 12-lead electrocardiogram revealed atrial fibrillation and complete atrioventricular block. (*A*) Chest radiography showed enlargement of heart with mild pulmonary congestion. (*B*).

The delivery sheath was introduced via the right femoral vein, and right ventriculography revealed marked right ventricular dilatation (*Figure 2*). The first two deployment attempts failed because of inadequate fixation and pacing capture (see Supplementary material online, *Video S1*). On the third attempt, although retrieval was attempted after failed pacing, (*Figure 3* and Supplementary material online, *Video S2*) tissue became interposed between the Micra device and the retrieval cup, preventing complete retraction (see Supplementary material online, *Video S3*). The device was subsequently removed by drawing it into the sheath. (*Video 1*) On gross inspection, tissue resembling tricuspid valve structures was noted between the device and the cup (*Figure 4*). The patient remained haemodynamically stable and asymptomatic during the procedure. A new Micra device was advanced to an appropriate position and implanted, but adequate pacing capture could not be achieved. The procedure was terminated with temporary pacing in place.

**Figure 2:**
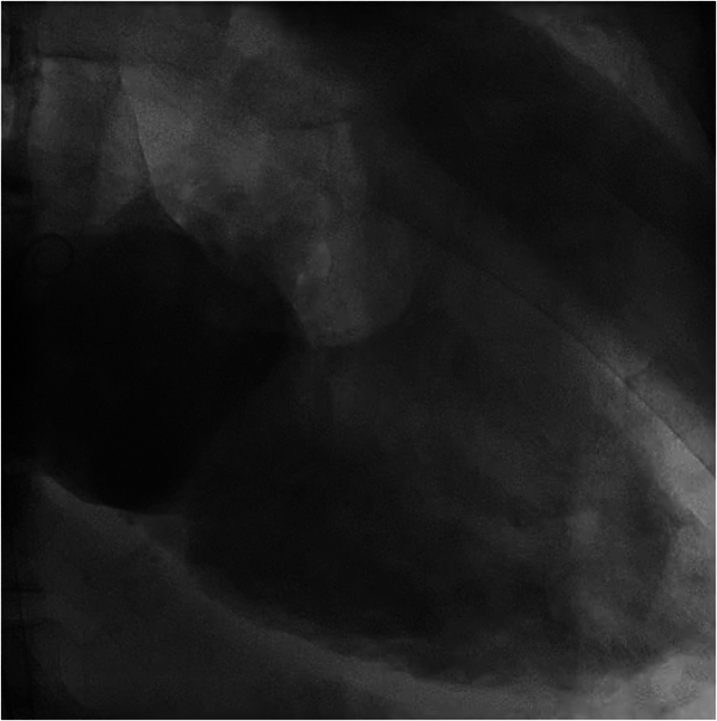
Angiography in right anterior oblique showed large right ventricle.

**Figure 3:**
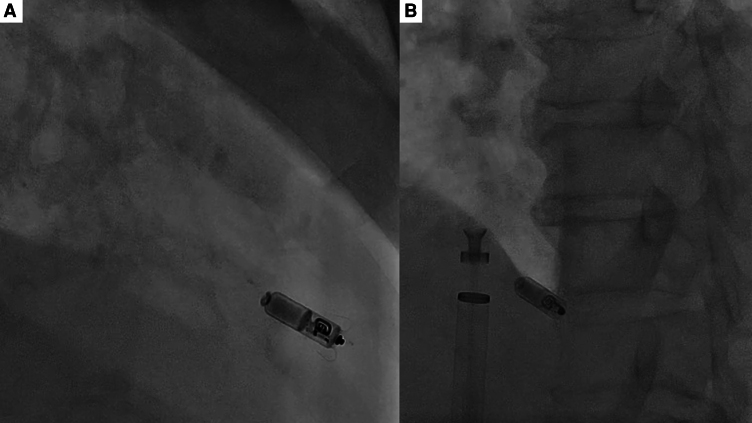
There are angiographies of RAO (*A*) and LAO (*B*) view on the third attempt.

**Figure 4:**
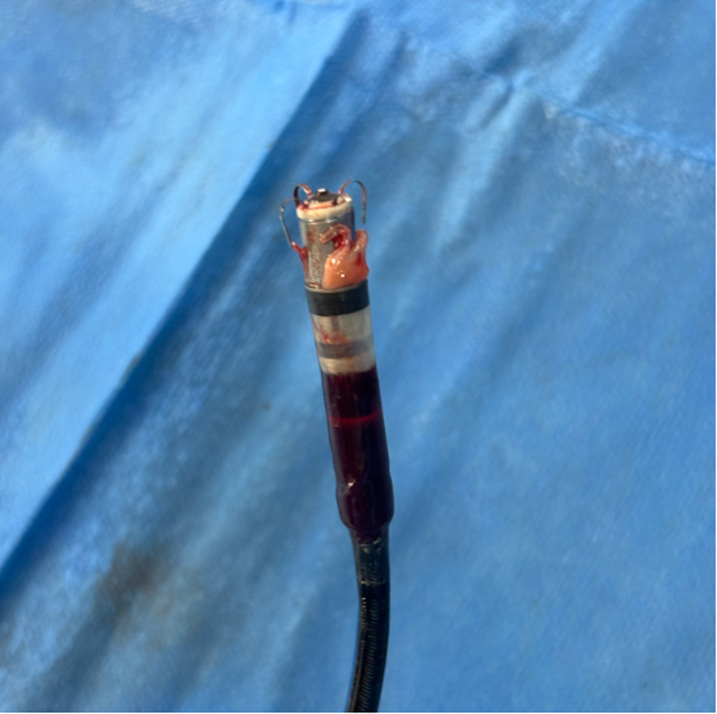
On gross inspection, tissue resembling tricuspid valve structures was noted between the device and the cup.

Postoperatively, the patient developed worsening of heart failure requiring non-invasive positive pressure ventilation (NPPV) and intravenous furosemide (20 mg twice daily). After intensive therapy, his respiratory status improved.

Subsequent TTE and transoesophageal echocardiography (TEE) demonstrated massive TR due to rupture of the anterior papillary muscle, which was prolapsing into the right atrium (*Videos 2* and *3* and Supplementary material online, *Videos S4–S6*).

In addition, transthoracic echocardiography performed after temporary pacemaker implantation continued to show severely reduced left ventricular systolic function, with an LVEF of 26.7%. Given that complete atrioventricular block would necessitate a high burden of ventricular pacing, and that P waves were present on the electrocardiogram, making atrioventricular synchrony through the addition of an atrial lead desirable. In addition, transthoracic echocardiography performed after temporary pacemaker implantation continued to show severely reduced left ventricular systolic function, with an LVEF of 26.7%. Given that complete atrioventricular block would necessitate a high burden of ventricular pacing, and that P waves were present on the electrocardiogram, making atrioventricular synchrony through the addition of an atrial lead desirable, the patient underwent Left Bundle Branch Optimized Cardiac Resynchronization Therapy (LOT-CRT) for preventing further declining cardiac function (*Figure 5*). Oral medications, including furosemide 20 mg and tolvaptan 3.75 mg once daily, were initiated. The patient’s respiratory symptoms improved, and neither pleural effusion nor leg oedema was observed.

**Figure 5:**
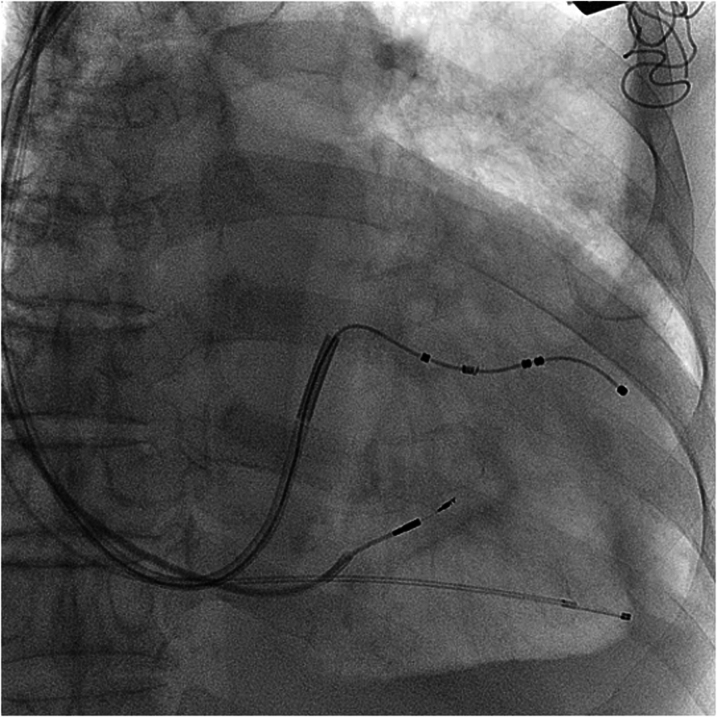
TV-PM with left bundle branch optimized cardiac resynchronization therapy implantation ended without any issues.

Surgical management was deemed inappropriate because of the patient’s frailty. Although transcatheter tricuspid edge-to-edge repair has since been approved, the gap between the anterior and septal leaflets exceeds 30 mm, making edge-to-edge repair technically challenging. Transcatheter valve implantation may be considered as a potential option; however, it remains unapproved, making clinical decision-making difficult. Therefore, the patient was managed with intensified oral diuretic therapy. Finally, he was transferred to a rehabilitation hospital under NYHA class1 status.

## Discussion

We present a case of TR resulting from tricuspid valve injury during LPM implantation. The clinical course of this patient suggested two important clinical issues.

First, TR may occur due to papillary muscle injury during LPM retrieval. Previous reports have documented TR resulting from mechanical interference after LPM implantation.^3,4^ Additionally, implantation of the LPM on the right ventricular (RV) septum has been associated with an increased risk of TR.^5,6^ However, reports of TR caused by papillary muscle injury at the time of implantation are still rare,^7^ and to our knowledge, papillary muscle rupture during device retrieval has not previously been reported.

Second, proper patient selection is crucial when considering LPM implantation. Although LPM, like TV-PMI, has been reported to worsen TR in the chronic phase, the mechanism by which it causes severe acute TR has not been well described.^6^ With respect to acute TR, a mechanism involving chordal injury after multiple deployment attempts has been reported and appears similar to the present case.^7^ Therefore, repeated deployment attempts may carry a risk of tricuspid valve injury. In addition, CT-based analyses have identified characteristics of cases in which LPM implantation is difficult,^8^ suggesting that preprocedural risk assessment may be important for avoiding this complication.

In this case, the LPM’s cup and body entrapped the anterior papillary muscle during implantation in the RV septum. During retrieval, the entrapped muscle interfered with retraction into the delivery sheath, necessitating direct recapture into the Micra sheath while pulling the tether. This manoeuvre resulted in rupture of the anterior papillary muscle (*Figure 4*), ultimately leading to significant TR.

In this case, we thought that the anterior papillary muscle, which is typically the largest and supports the anterior and posterior leaflets of the tricuspid valve,^9^ became entrapped between the delivery sheath and the leadless pacemaker during deployment, resulting in its rupture and subsequent massive tricuspid regurgitation. TEE clearly demonstrated the anterior papillary muscle prolapsing into the right atrium, along with abnormal motion of both the anterior and septal leaflets.

Given the significant chamber enlargement, the risk of complications associated with LPM implantation was considered high in this patient. Under this situation, the jugular vein approach may avoid the risk of complications.^10^

## Conclusion

Papillary muscle entrapment and rupture during leadless pacemaker retrieval represent a rare but catastrophic mechanism of tricuspid regurgitation. Operators should be aware of this potential complication, particularly in patients with a significantly dilated right-sided chamber.

## Supplementary Material

ytag418_Supplementary_Data

## Data Availability

The datasets used and/or analysed during the current study are available from the corresponding author on reasonable request.

## Lead author biography

Hikaru Tanemura is a cardiac and peripheral intervention specialist at Shonan Kamakura General Hospital in Japan. He received his medical degree at the Toho University in 2021.

## Notes

**Consent:** Informed consent was obtained from the patient in accordance with the Committee on Publication Ethics (COPE) guidelines.
